# Sneddon’s syndrome: A case report with diagnostic approach and imaging review

**DOI:** 10.1016/j.radcr.2025.06.041

**Published:** 2025-07-09

**Authors:** Soukaina Beyyato, Zineb Yammouri, Hajar Ouazzani, Ismail Chaouche, Amal Akammar, Nizar El Bouardi, Meriem Haloua, Moulay Youssef Alaoui Lamrani, Mustapha Maaroufi, Meryem Boubbou, Badreeddine Alami

**Affiliations:** a Department of Radiology and Interventional Imaging, CHU Hassan II, FEZ, Sidi Mohammed Ben Abdellah University, Fes, Morocco; b Department of Radiology Mother and Child and Interventional Imaging, CHU Hassan II, FEZ, Sidi Mohammed Ben Abdellah University, Fes, Morocco

**Keywords:** Sneddon syndrome, Ischemic strokes, Livedo racemosa, MRI

## Abstract

Sneddon’s syndrome is a rare thrombotic vasculopathy characterized by the association of livedo racemosa and ischemic cerebrovascular events, typically affecting young to middle-aged women. We report the case of a 41-year-old woman who presented with sudden right-sided hemiparesis, expressive aphasia, and a history of livedo racemosa. MRI revealed bilateral ischemic lesions and cortical atrophy, while autoimmune and thrombophilia workups were negative. This case emphasizes the importance of considering Sneddon’s syndrome in young patients with recurrent strokes and livedo racemosa, and highlights the diagnostic value of MRI combined with clinical evaluation.

## Introduction

Sneddon’s syndrome is a rare noninflammatory arteriopathy characterized by thrombotic occlusion of small and medium-sized arteries. Its etiology remains unclear, though proposed mechanisms include autoimmune responses, endothelial dysfunction, and inherited or acquired thrombophilia. A notable association with antiphospholipid antibodies exists in approximately half of cases.

The syndrome primarily affects young to middle-aged adults, especially women between the ages of 30 and 50. Clinically, Sneddon’s syndrome presents with recurrent ischemic strokes or transient ischemic attacks (TIAs), cognitive decline, and livedo racemosa persistent, violaceous, net-like skin discoloration that often precedes neurological symptoms by several years.

Diagnosis is based on clinical findings and is supported by dermatologic examination, brain MRI, and sometimes skin biopsy. Neuroimaging typically reveals multifocal ischemic lesions and cortical atrophy, while laboratory workup may assist in excluding other autoimmune or thrombotic disorders.

## Case presentation

A 41-year-old woman presented to the neurology department with sudden-onset right-sided hemiparesis and expressive aphasia. Her past medical history included rheumatic mitral valve stenosis and a 6-year history of unexplored livedo racemosa. She also reported multiple episodes of transient ischemic attacks over the past 2 years, which had not been further investigated. There was no history of migraine, seizures, or visual disturbances. Her surgical history was unremarkable. Family history was negative for autoimmune diseases, strokes, or thrombotic disorders.

On admission, her vital signs were stable: blood pressure 130/80 mmHg, heart rate 78 bpm, respiratory rate 16 breaths/min, temperature 36.8°C, and oxygen saturation 98% on room air. Neurological examination confirmed right hemiparesis and expressive aphasia. Dermatological examination showed widespread erythematous-violaceous reticular lesions on the arms, legs, and trunk, consistent with livedo racemosa.

Initial laboratory investigations were within normal limits: hemoglobin 13.4 g/dL, white blood cell count 7200/mm³, platelet count 250,000/mm³, C-reactive protein 2.5 mg/L, ESR 12 mm/h. Renal and liver function tests were normal. Autoimmune screening revealed negative antinuclear antibodies, antiphospholipid antibodies (anticardiolipin IgG/IgM and lupus anticoagulant), and negative anti-dsDNA and ANCA.

Cerebral MRI showed multiple bilateral ischemic lesions predominantly involving the frontal and parietal lobes, along with cortical atrophy. Transthoracic echocardiography revealed moderate mitral valve stenosis with no intracardiac thrombi. Cerebral angiography was not performed due to the absence of clinical suspicion of vasculitis and normal inflammatory markers.

Given the presence of recurrent cerebrovascular events, livedo racemosa, and a negative autoimmune and thrombophilia work-up, a clinical diagnosis of idiopathic Sneddon’s syndrome was established after exclusion of other differential diagnoses, including systemic lupus erythematosus, vasculitis, and antiphospholipid syndrome.

**Fig. 1:**
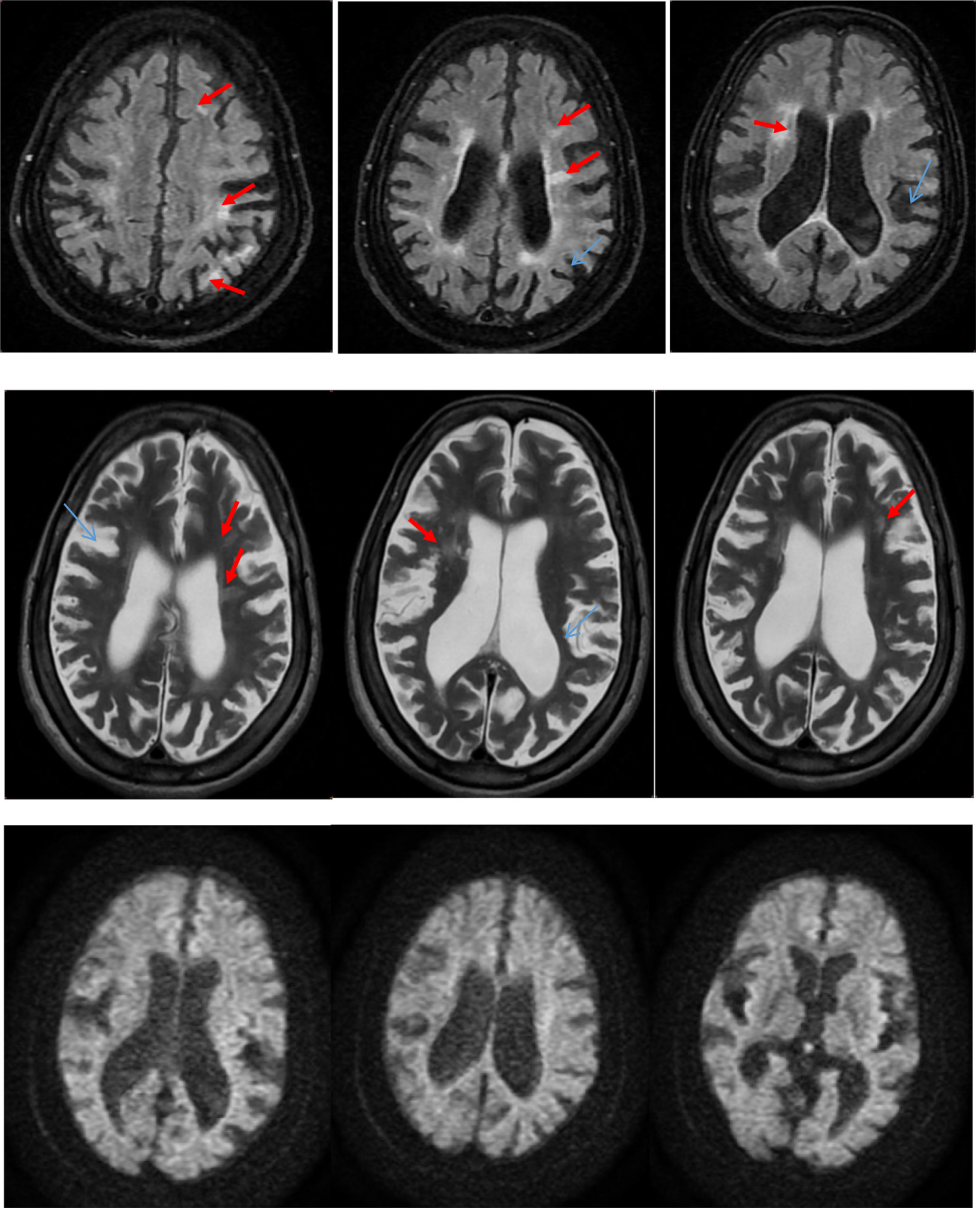
Axial MRI FLAIR, T2, and diffusion sequences showing multiple hyperintense areas in the periventricular deep white matter and bilateral frontal and parietal territories (red arrows), without diffusion restriction, and associated with cortical atrophy (blue arrows).

The patient was started on long-term antiplatelet therapy (aspirin 160 mg/day) and secondary stroke prevention measures, including statins and blood pressure control. Given the absence of antiphospholipid antibodies, anticoagulation was not initiated. Over the following weeks, she showed gradual improvement in motor function with persistent mild aphasia. She was discharged to a neurorehabilitation facility and continued to improve over a 3-month follow-up, with no recurrence of stroke or new symptoms (Fig. 1).

## Discussion

Sneddon’s syndrome was first described by Ian Sneddon in 1965 as a clinical entity involving livedo reticularis and cerebrovascular disease. It is a progressive arteriopathy affecting small and medium-sized arteries, resulting in both cutaneous and central nervous system involvement [1,2].

Three subtypes have been proposed: idiopathic (not associated with SLE or antiphospholipid antibodies), primary antiphospholipid syndrome-related, and secondary to systemic lupus erythematosus [3]. The pathogenesis remains unclear but may involve microvascular thrombosis and immune-mediated endothelial injury. Genetic predisposition has been suggested in rare familial cases, and environmental factors such as smoking and oral contraceptive use may contribute to thrombotic risk [4,5].

Livedo racemosa is typically the earliest manifestation, often preceding neurologic symptoms by several years [2]. The skin lesions are violaceous, net-like, and persistent, most commonly involving the trunk and extremities. Neurological involvement may include ischemic strokes, TIAs, seizures, migraines, cognitive decline, and psychiatric symptoms [5]. Other possible systemic manifestations include renal involvement, cardiovascular abnormalities, and pregnancy-related complications such as fetal loss [6].

Laboratory findings are often nonspecific or normal. Immunologic studies are typically negative, although occasional patients may test positive for cryoglobulins, ANA, antiphospholipid antibodies, or lupus anticoagulant. A comprehensive laboratory workup is essential to rule out differential diagnoses [1,[6], [7], [8]].

MRI is the imaging modality of choice, revealing multiple ischemic infarcts, particularly in the subcortical white matter and basal ganglia, with or without cortical atrophy [2]. CT scans are less sensitive, particularly in the early stages. In our patient, MRI findings were consistent with typical features of Sneddon’s syndrome.

There is no definitive treatment [9]. Management is primarily preventive, focusing on reducing thrombotic risk. Antiplatelet agents and anticoagulants are commonly used, especially in cases associated with antiphospholipid antibodies [9,10]. Corticosteroids and immunosuppressants have not shown consistent benefits. Lifestyle modifications, including smoking cessation and avoidance of estrogen-containing contraceptives, are recommended [11].

## Conclusion

Sneddon’s syndrome is a rare but important differential diagnosis in young patients with recurrent strokes and livedo racemosa. Early recognition and appropriate management are crucial to prevent further neurological decline. MRI plays an important role in diagnosis, and a multidisciplinary approach is often necessary to rule out other systemic diseases. This case highlights the importance of considering Sneddon’s syndrome in appropriate clinical settings to facilitate timely diagnosis and management.

## Patient consent

I, the author of the article “Sneddon’s syndrome: A Case Report with Diagnostic Approach and Imaging Review” declare that informed written consent was obtained from the patient for publication of the Case Report and all imaging studies in RADIOLOGY CASE REPORTS.
